# Intuitive Logic Revisited: New Data and a Bayesian Mixed Model Meta-Analysis

**DOI:** 10.1371/journal.pone.0094223

**Published:** 2014-04-22

**Authors:** Henrik Singmann, Karl Christoph Klauer, David Kellen

**Affiliations:** Institut für Psychologie, Albert-Ludwigs-Universität Freiburg, Freiburg, Germany; Brain and Spine Institute (ICM), France

## Abstract

Recent research on syllogistic reasoning suggests that the logical status (valid vs. invalid) of even difficult syllogisms can be intuitively detected via differences in conceptual fluency between logically valid and invalid syllogisms when participants are asked to rate how much they like a conclusion following from a syllogism (Morsanyi & Handley, 2012). These claims of an intuitive logic are at odds with most theories on syllogistic reasoning which posit that detecting the logical status of difficult syllogisms requires effortful and deliberate cognitive processes. We present new data replicating the effects reported by Morsanyi and Handley, but show that this effect is eliminated when controlling for a possible confound in terms of conclusion content. Additionally, we reanalyze three studies (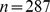) without this confound with a Bayesian mixed model meta-analysis (i.e., controlling for participant and item effects) which provides evidence for the null-hypothesis and against Morsanyi and Handley's claim.

## Introduction

Decades of research have come to a relatively unanimous conclusion on the role of formal logic in human reasoning: Naive reasoners (i.e., reasoners untrained in formal logic) make many errors when asked to respond to reasoning problems in accordance with the norms of formal logic. Furthermore, other factors such as the content and context may prompt what is known as a *heuristic* response [1]–[4]. Naive reasoners' deviations from formal logic have played a major role in some of the most successful research programs within cognitive psychology [5] and have led to a variety of new paradigms which abandon classical bivalent logic as their normative yardstick [6]–[8].

One prominent finding demonstrating people's limited ability to act in accordance with formal logic is the *belief bias* effect in syllogistic reasoning [9], [10]. Syllogisms are arguments consisting of two premises and one conclusion such as the following two examples (which contain a non-word as the middle term):

*Example 1:*

No ice creams are vons.

Some vons are hot.

Therefore, some ice creams are not hot.

*Example 2:*

No expensive things are mets.

Some mets are diamonds.

Therefore, some diamonds are inexpensive.

When asked to evaluate whether or not the conclusion follows with logical necessity, individuals usually show the following response pattern [9]: they are more likely to accept a believable but invalid conclusion (Example 1) than a valid but unbelievable conclusion (Example 2). Resisting the temptation to give the heuristic response (i.e., responding in line with a conclusion's believability) is correlated with people's general cognitive ability [11]. Furthermore, when asked to generate a valid conclusion from premises of the form in the examples (with abstract material), only 13% of participants are able to do so correctly ([12], Syllogism IE2, Table 7). Taken together, these and other findings converge on the conclusion that evaluating the validity of such syllogisms is a resource-demanding and effortful cognitive process that requires goal-directed manipulation and coordination of multiple mental representations (see [12] for an overview of the relevant theories).

According to the view sketched above, formal logic is not deeply entrenched in individuals' cognitive systems. In contrast, recent theoretical developments revive the idea of classical logic as part of the human cognitive system and postulate that individuals possess *logical intuition*. In particular, de Neys [13] and Morsanyi and Handley [14] propose that even when giving the formally incorrect response (e.g., accepting the conclusion in Example 1) individuals intuitively detect the conflict between the normatively correct solution and the heuristic response. This conflict detection is thought to be “automatic” and “implicit” ([13], p. 30) and “does not seem to be conscious” ([14], p. 597). Proponents of logical intuitions argue that such intuitions are revealed by indirect means such as physiological measures, response times or indirect tasks (e.g., longer response times for trials in which the normative and heuristic responses are in conflict with each other [15]).

In this paper we focus on the recent work by Morsanyi and Handley [14] (henceforth referred to as MH). Their hypothesis of a *fluency mediated intuitive logic* starts from the notion sketched above that, when reading syllogisms like Examples 1 and 2, individuals can intuitively detect the true logical status of the syllogisms. In order to measure this intuitive sense of logicality, MH adapt ideas and methods from the literature on fluency effects [16]–[19]. Their hypothesis can be broken down into two steps.

First, MH assume that when reading the premises of a (sequentially presented) syllogism, individuals construct a (initial and implicit) mental model of the syllogism. When finally the conclusion is presented this can have two different consequences: a valid conclusion is processed with higher conceptual fluency as it matches the initial model, whereas an invalid conclusion is processed with comparatively low fluency as it need not match the initial model. MH's intuitive logic rests on this difference in perceived processing or conceptual fluency in an argument that is constructed in analogy to arguments for sequentially presented word triads (where a coherent word triad, e.g., salty - deep - foam, elicits higher fluency than an incoherent triad, e.g., dream - ball - book [16]).

The second part of MH's argument is based on the *hedonic-marker hypothesis* [18] according to which higher fluency triggers positive affect. Consequently, MH expected valid syllogisms to elicit more positive affect than invalid syllogisms. They tested this hypothesis in a straightforward manner by presenting syllogisms and asking participants to judge the likability of the conclusion on a 5-point scale ranging from “Don't like it at all” to “Like it very much”. In four experiments MH found evidence for their hypothesis (i.e., higher liking ratings for valid than for invalid syllogisms) using different syllogisms and experimental manipulations.

As the idea of intuitive logic is a relatively new theoretical development that stands in contrast with more established theories on human reasoning, Klauer and Singmann [20] (henceforth referred to as KS) tested alternative accounts for MH's findings based on certain characteristics of MH's experimental design. In particular, KS noted that MH did not randomly assign the validity of the syllogisms to the different conclusions used in their study. For example, the conclusion of Example 1 (“Therefore, some ice creams are not hot”) was only presented as part of the invalid syllogism given in Example 1 and not in a valid syllogism such as the following ([12], Syllogism IE4, Table 7):

*Example 3:*

No hot things are vons.

Some vons are ice creams.

Therefore, some ice creams are not hot.

The fact that each conclusion was not randomly assigned to valid and invalid syllogisms but had a fixed validity status opens the possibility that the findings of MH are solely due to the affective connotations of the conclusions and not related to the validity of the syllogism. In a series of experiments KS found that when experimentally controlling for this possible confound by randomly assigning the different contents (i.e., conclusions of the syllogisms) to valid and invalid syllogisms, the supposed effects of validity on liking ratings disappeared. In a first step they only presented the conclusions of the conditionals without the premises (participants were simply asked to indicate how much they liked, e.g., “Some ice creams are not hot” or “Some diamonds are inexpensive”) and found the exact same pattern of responses as obtained with the full syllogisms including the original (non-randomized) premises (see Experiment 2 by KS). This strongly suggested that the effect obtained by MH was mainly driven by the specific content of the conclusions and their affective connotations.

In two further experiments, KS presented participants with syllogisms in which the conclusions remained fixed but the validity of the syllogisms was randomly assigned by altering the form of the premises (e.g., one participant saw Example 1 whereas another participant saw Example 3). When conclusions were equally likely to be either presented as part of a valid or an invalid syllogism, the results showed no evidence for an influence of the validity of the syllogism on participants' liking ratings (Experiments 3 and 4 by KS). These results questioned the idea of a fluency mediated intuitive logic as proposed by MH.

Despite considerable statistical power, the experiments by KS were somewhat incomplete with regard to one important finding: They did not replicate the original MH validity effect on liking ratings. Instead they found an interaction of validity and believability, discussed in more detail below. This failure was attributed to potential differences in the subtle affective connotations of the materials when presented in English to native English speakers (as done by MH) as compared to when presented in German to native German speakers (as done by KS). One additional shortcoming was that KS only employed one of the experimental procedures employed by MH, which may have been the one most adverse to finding effects of an intuitive logic.

The remainder of this paper is split into two parts. In the first part we present new data obtained with the original MH material presented to native English speakers using an experimental paradigm of MH not employed by KS in which we replicate the original MH validity effect. However, we show that this effect again disappears when controlling for conclusion content by randomly assigning the different conclusions to either valid or invalid syllogisms. In the second part, we reevaluate the evidence for an intuitive logic gathered within the paradigm developed by MH with a meta-analysis based on a Bayesian ANOVA [21] which we present and discuss in detail.

## Experiments 1 and 2

In contrast to MH, KS did not find main effects of validity and believability when presenting the (translated to German) syllogisms without randomization of conclusions across validity status, but an interaction of validity and believability. The validity effect appeared in the expected direction only for the unbelievable conclusions and the reversed pattern was found for believable conclusions. Although not a direct replication of MH's results pattern, these findings could be interpreted as an effect of validity on liking ratings. However, KS introduced a condition with *randomized* conclusions in which, by reordering the terms in the premises, each conclusion could appear as a valid or invalid syllogism. In this condition KS found no evidence of an effect of validity, but only a main effect of believability (higher liking ratings for believable conclusions).

In their effort to test the hypothesis of a fluency mediated intuitive logic, KS replicated (with modifications) MH's Experiment 4, which both MH and KS considered to be the strongest test of this hypothesis based on a number of features of its procedures: Specifically, whereas in MH's Experiments 1 to 3 the sequential presentation of the syllogism was self-paced (i.e., participants could decide when they would view the next part of the syllogism) this was not the case in MH's Experiment 4 and KS (i.e., each statements remained on the screen for a given time, for example for 2 seconds, then disappeared and the next statement appeared automatically). Additionally, in MH's Experiments 1 to 3 the second premise remained on the screen along with the conclusion when giving the liking ratings and the conclusions were prefaced with the word “therefore” (which provides a strong linguistic marker indicating that the three statements are linked in an inferential context). This was not the case in MH's Experiment 4 and KS. In sum, the variations implemented in MH's Experiment 4 and KS removed the inferential reasoning context from the task participants faced and removed the self-paced nature of the argument presentation. However, it may well be possible that without any reference to the inferential context or with a fast-paced presentation schedule, the intuitive logic may not be “activated” and hence either of these variations may have prevented a genuine effect of validity.

The purpose of Experiments 1 and 2 was (a) to test whether we can replicate MH's main effect for validity in a sample of English speakers and (b) whether more compelling evidence for an intuitive logic can be found when providing a context that more strongly cues inferential reasoning. In the present experiments we therefore employed the procedures used in MH's Experiment 2 in a web-based experiment using the original English material. In Experiment 1 we replicated MH's Experiment 2 by using the material with fixed validity. In Experiment 2 we presented the conclusions with randomized validity. We only used the syllogisms with believable and unbelievable conclusions (i.e., we omitted the conditionals with “abstract” conclusions also used by MH and KS).

### Method

#### Ethics Statement

The ethical principles as formulated in the WMA Declaration of Helsinki guided our research project. If research objectives do not refer to issues regulated by law (e.g., the German Medicine Act [Arzneimittelgesetz, AMG], the Medical Devices Act [Medizinproduktegesetz, MGP], the Stem Cell Research Act [Stammzellenforschungsgesetz, StFG] or the Medical Association's Professional Code of Conduct [Berufsordnung der Ärzte]), then no ethics approval is required for social science research in Germany. Our study has no such objectives; therefore, no approval was required. Participation was entirely voluntary, data were collected anonymously over the internet and could only be accessed by the first author and subjects were fully informed about the study purpose and their anonymitiy before proceeding to answer the questionnaire. As the questionnaire could reasonably be assumed not to cause subjects any harm or distress, written consent was not obtained but subjects' decision to participate was considered to imply their consent. This procedure was in accordance with the German Society for Psychology's research standards (Grundsätze der Forschung am Menschen, C.III, para. 6).

#### Participants

Participants were recruited via CrowdFlower.com (a service similar to Mechanical Turk) and had to live within the UK (which was ensured via IP filtering). Twenty-six participants completed Experiment 1 (three additional participants did not finish the experiment). Of those, we excluded four participants who indicated that English was not their first language. The remaining twenty-two participants had a mean age of 28 years (SD = 9.02, ranging from 16 to 50 years) and came from diverse background (two high school students, four university students, eleven employees/working, and five unemployed/homemaker).

Sixty-nine participants completed Experiment 2 (nine additional participants did not finish the experiment). Of those, we excluded twelve participants who indicated that English was not their first language. The remaining fifty-seven participants had a mean age of 34.1 years (SD = 10.61, ranging from 18 to 71) and came from diverse background (five university students, thirty-nine employees/working, and seventeen unemployed/homemaker or “other”). All participants received a small monetary compensation for participation.

#### Materials and Procedure

We used the 8 believable and 8 unbelievable syllogisms from MH's Experiment 2 in the present Experiment 1 and versions of those syllogisms in which we randomized the validity status of the conclusions in the present Experiment 2 (as in the above Examples 1 and 3; see KS, Experiment 3, for more details). The procedure otherwise closely followed MH's Experiment 2 (normal font condition, only liking ratings): For each syllogism, the first premise was presented first. After clicking on “Next”, the first premise disappeared and the second premise appeared. After clicking on “Next” again, the conclusion appeared including “Therefore” as first word (while the second premise remained on the screen) and participants had to indicate how much they liked the final statement by clicking on one of five smileys/sad faces arranged in a 5-point scale ranging from “Don't like it at all” to “Like it very much”. The items were presented in randomized order and the randomization (including the randomization of conclusion validity in Experiment 2) was done anew for each participant. We presented an additional warm-up syllogism based on a different content prior to the 16 experimental syllogisms, which was not analyzed. Each experiment implemented a within-subjects design with two factors: believability (believable and unbelievable) and validity (valid and invalid).

### Results and Discussion

**Figure 1:**
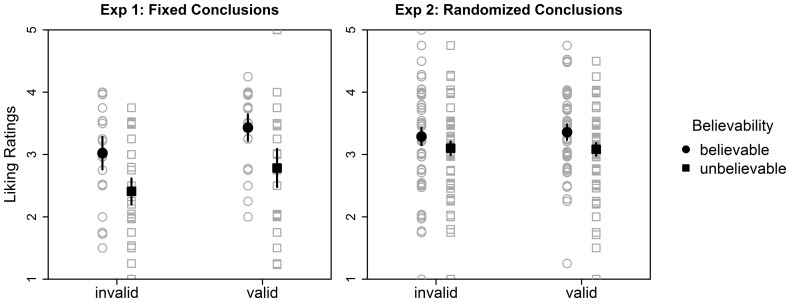
Mean (filled symbols) and individual (nonfilled symbols) liking ratings in Experiments 1 (left panel) and 2 (right panel) as a function of validity and conclusion believability. A small amount of vertical jitter was added to individual liking ratings to avoid perfect overlap of two ratings. Error bars show difference adjusted 95% Cousineau-Morey confidence intervals for within-subject designs (i.e., non-overlapping error bars indicate significant differences [58]).

For each experiment we analyzed the data using a separate within-subjects ANOVA with factors believability and validity. (When analyzing both data sets together, a significant interaction of validity and experiment emerged, 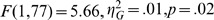. Note, 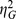 (generalized eta-squared) is the recommended effect size for repeated measures designs according to [22].) Results are displayed in Figure 1.

#### Experiment 1

When validity was not randomly assigned to the different conclusions we replicated MH's main findings. We found a main effect of validity, 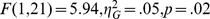, indicating that participants gave higher ratings to valid than invalid syllogisms. Additionally, we found a main effect for believability, 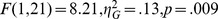, indicating that participants gave higher liking ratings to believable than to unbelievable conclusions. The interaction of validity and believability did not reach significance, 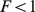.

#### Experiment 2

In line with KS, we did not find an effect of validity, nor an interaction of validity with believability when randomly assigning validity to the different conclusions, both 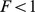. However, the main effect of believability (higher liking ratings for believable than unbelievable conclusions) was significant, 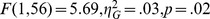.

#### Discussion

The results are relatively clear cut. When replicating MH with native English speakers their results were replicated, including the main effect of validity that KS did not replicate: There were higher liking ratings of valid than invalid conclusions. However, interpreting these results as evidence for an intuitive logic again seems premature. When controlling for possible effects of content by randomly assigning the different conclusions to either valid or invalid syllogisms (Experiment 2), the validity effect disappears. This strongly indicates that the procedural variations implemented in MH's Experiment 4 and KS compared to MH's Experiments 1 to 3 were not responsible for the failure to replicate evidence for an intuitive logic. Furthermore, the fact that we could replicate the original validity main effect, whereas KS found interactions of validity and believability, supports the assumption that differences in cultural norms and subtle affective connotations to the material between English and German participants influence the liking ratings (this is only apparent in the data when non-randomly assigning conclusion validity).

## A Bayesian Mixed Model Meta-Analysis

The experiments by KS and our new data have painted a relatively pessimistic picture regarding MH's claims of a fluency mediated intuitive logic. When controlling for content effects by randomly assigning the conclusions to valid or invalid syllogisms, there was no evidence for this hypothesis. However, there are also reasons why abandoning MH's idea of a fluency mediated intuitive logic may be premature.

First, the notion of an intuitive logic is theoretically interesting as it is one of many recent approaches dealing with the question of how analytic and heuristic processes interact to come to a single response in a given reasoning situation [23]–[25]. Given that it is one of those approaches that runs counter more mainstream theories of reasoning, it runs the risk of being discounted just because of having an outsider status. Additionally, proponents cite a range of evidence that is not addressed by the current discussion ([13] for an overview) and support MH's ideas of an intuitive logic even after KS published their results [25], [26].

Second, providing evidence against the notion of an intuitive logic as proposed by MH in the framework of null-hypothesis significance testing (NHST) is difficult [27], because NHST can provide evidence against a null hypothesis more compellingly than evidence in favor of a null hypothesis as was attempted here. Only relatively recently have alternatives to NHST been proposed that, based on Bayesian statistics, can provide evidence in favor of the null hypothesis [28]. An additional statistical issue arises from KS' and our findings that the content of the conclusions plays a major role in participants' liking ratings. Ignoring these item effects by simply averaging across items can also severely distort the results [29], an issue long known in psycholinguistics [30]. Given these methodological critics and the intriguing claims of an intuitive logic, it seems appropriate to reanalyze the data gathered so far using the most up-to-date statistical methodology. Additionally, analyzing all data sets together should uncover even very small effects.

### Bayesian Statistics

Although many of the problems surrounding the use of null-hypothesis significance testing (NHST) and 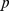-values have been known for a long time [27], [31], [32], their discussion has been rekindled due to the recent availability of alternative statistical methods [28], [33]. One of the core criticisms against NHST concerns the fact that 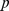-values can only state evidence *against* the established null hypothesis in cases like the one addressed here. This severely constrains the type of inferences that can be made from data given that accepting the null hypothesis only means that there is a lack of evidence against the null but does not imply that there is evidence in favor of it. One immediate consequence of this asymmetry in NHST is that it does not support the testing of invariances, invariances which in many cases represent core properties of scientific theories (e.g., [34]). In the present case, theories that do not postulate the existence of intuitive logic predict an invariance for the liking ratings of valid and invalid syllogisms.

Moreover, NHST is also biased towards overstating the evidence against the null hypothesis, leading researchers to reject the latter in cases where there is actually very little evidence against it. For example, Wetzels, Matzke, Lee, Rouder, Iverson, & Wagenmakers [35] showed that several 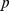-values between.05 and.01 reported in the literature actually correspond to cases where evidence against the null hypothesis was little more than anecdotal.

A principled alternative to NHST can be found in Bayesian modeling [36], a framework that allows for the quantification of evidence in favour of different hypotheses. The core principle underlying statistical inference in the Bayesian framework is Bayes' theorem, which indicates how prior beliefs towards different models or hypotheses (the two terms are interchangeable) can be updated on the basis of observed evidence. In the Bayesian framework, beliefs can be expressed by means of probabilities.

Let 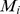 denote a model among a set of 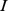 candidate models, 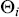 the set of parameters in model 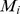. Given that the present paper is focused on ANOVA-type models, parameters correspond to the different effects that decompose data from a particular experimental design such as main effects or interactions [37]. Finally, let 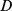 denote the observed data. According to Bayes' theorem, the (posterior) probability of model 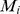 given the observed data D is given by:
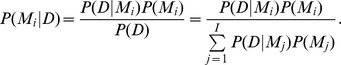



The first term in the numerator corresponds to the so-called *marginal likelihood*, the likelihood of the data according to model 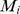 when integrating across the latter's parameter space 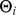, so that
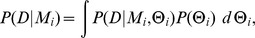
where P(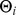) denotes the *prior probability* (densities) associated to the model's parameter space. 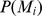 denotes the prior probability of model 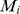, which will be for all practical purposes ignored, as discussed below. Prior 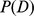 corresponds to the probability of the observed data, which according to the *Law of Total Probability* corresponds to the weighted sum of each model's marginal likelihood.

The marginal likelihood's integration across the model's parameter space corresponds to an evaluation of the likelihood of the data across data patterns in which data that are consistent with the model receive high weights. For example, consider an idealized dataset where all effects across the factorial design are perfectly additive (i.e., there are no interactions). If you consider the likelihood of this dataset across an ANOVA-model that includes interactions, the data will be very unlikely in many regions of the parameter space where interaction effects are assumed (i.e., the likelihood will be low in these regions). In comparison, larger likelihoods are obtained across the parameter space of a model assuming no interactions, leading to a larger marginal likelihood. The prior 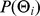 determines the weight given to each region of the parameter space; for example if the prior reflects the expectation of small effects then a greater weight will be given to the likelihoods correspoding to regions of the parameter space where small effects are assumed. Taken together, the marginal likelihood of a model is not only affected by its ability to match the observed data pattern but also by the match between the observed data and the model's whole range of predictions, a range that is weighted by the parameter priors. This has important consequences in terms of model selection as discussed below.

The representation of the posterior probabilities of two models, let us say 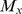 and 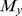, in terms of *posterior odds* provides a convenient way of comparison:
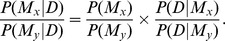



The first term on the right side of the equation corresponds to the models' prior odds, while the second term, known as the *Bayes factor* (BF) [38], quantifies the relative evidence for each model. The Bayes factor can be interpreted as the change from prior odds to posterior odds that is brought about by the observed data. For example, BF_*x,y*_ = 10 indicates that the posterior odds are shifted by a factor of 10 towards model 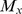 upon having obtained the data. Table 1 provides some guidelines on how to interpret Bayes factors. Furthermore, Bayes factors provide a quantification of evidence that is consistent across model comparisons. For example, if the Bayes factor for models 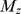 and 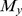 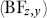 is 20, then the Bayes factor for models 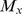 and 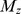 is 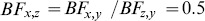. In other words, Bayes factors can be compared across models.

**Table 1:** Evidence Categories for the Bayes Factor.

Evidence	BF_*x,y*_	log(BF_*x,y*_)
Extreme (for 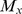 )	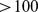	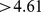
Very strong (for 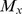 )	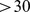	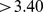
Strong (for 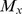 )	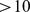	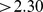
Substantial (for 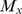 )	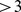	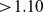
Anecdotal (for 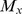 )	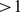	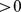
none	1	0
Anecdotal (for 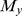 )	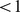	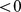
Substantial (for 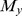 )	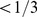	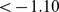
Strong (for 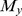 )	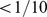	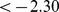
Very strong (for 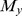 )	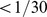	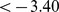
Extreme (for 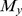 )	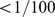	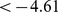

*Note.* Evidence categories as introduced by Jeffreys [59] (as cited by [40]).

Bayes factors quantify the evidence for each model independent of each model's prior odds, which can radically differ depending on the nature of the problem being discussed. For example, in the present discussion one could argue that the hypothesis of intuitive logic is less likely a priori than the hypothesis that there is no such thing as intuitive logic (given that it does not follow from any major theory of reasoning). Others could neverthless attempt to make the opposite case and argue that intuitive logic is very likely. Because Bayes factors quantify the change in evidence brought about by the data, irrespective of each model's prior odds, one can sidestep this debate and the inevitable subjectiveness associated to it.

Although Bayes factors are independent of each models' prior odds, they are sensitive to each model's range of predictions as well as to the parameter priors. The Bayes factor penalizes models that are able to account for unobserved data patterns: For example, the marginal likelihood of the above-mentioned idealized dataset is larger under a model that only assumes main effects than under a model that assumes interactions as well. This characteristic of Bayes factors indicates that they naturally penalize more complex and flexible models, especially when that flexibility does not make any substative contribution. This penalization embodies a principle of parsimony also known as Occam's Razor [39], [40].

The same principle of parsimony applies to the case of parameter priors: Let us assume that the main effects in the idealized dataset are small. The Bayes factor will penalize models with priors that do not incorporate any expectations regarding the size of the effect (e.g., completely non-informed priors assuming that large effects are as probable as small effects) in comparison to models with priors that reflect the small size of the effects. The impact of parameter priors is particularly relevant when testing a small effect against a null hypothesis given that an uninformed prior will reduce to a certain extent the relative evidence in favour of an effect.

In terms of traditional hypothesis testing, both the null and alternative hypotheses correspond to models (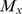 and 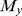, respectively) for which evidence is quantified. In contrast with 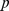-values, Bayes factors can provide both evidence against and in favor of the null hypothesis. Rouder and colleagues [21] provided algorithms for computing Bayes factors in ANOVA designs. These algorithms are currently available in the BayesFactor package [41] for the statistical programming language R [42].

### Participant and Item Effects

Another important addition to the statistical toolkit are *mixed models* (also known as mixed-effects, hierarchical, or multilevel models) [29], [43], [44] which allow for what is known as *crossed random effects*. A random effect or factor is often understood as a factor whose levels are considered to be sampled from a larger population with the intention to generalize across all levels of the factor. Classical statistical procedures based on the general linear model, such as ANOVA, can only handle one random effect, usually the participants (i.e., one tries to generalize across participants).

However, often one can also view items as random factors. For example, in research on syllogisms one usually does not want to make inferences on only the specific syllogisms studied but on the population of all syllogisms. To be able to do so requires the researcher to treat stimuli as random effects as well. Traditionally, this was only possible by performing two separate analysis for participants and items (i.e., what is known as the 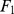 and 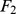 analysis; see e.g., [45] for an example in the reasoning domain). Only recently have procedures allowing for simultaneously analyzing multiple or crossed random effects, been made available [21], [43].

An important distinction in the literature on mixed models is between *random intercepts* and *random slopes* [46]. Random intercepts are usually added for all random factors and add a separate offset to the overall intercept (i.e., grand mean) for each level of a random factor and thereby capture the variance that is associated with the overall heterogeneity of the different levels of a random factor. For example, random intercepts for participants estimate the deviation of each participant's mean response from the overall mean, thereby allowing for an individual mean for each participant that may deviate from the grand mean. Random slopes can be added for each factor that varies across the levels of a random factor: For participants, random slopes can be added for within-subjects factors. For items, random slopes can be added for those factors that vary across the items (e.g., validity when it is randomized across items). Random slopes add a separate offset or deviation to the overall fixed effect for each level of the random factor and capture the variance that is associated with the heterogeneity of the fixed effect across levels of the random factor. For example, random slopes for believability for participants estimate the deviation of each participant's believability effect from the overall believability effect, thereby allowing for an individual believability effect of each participant. As all random effects only estimate offests or deviations from overall effects, they are usually assumed to be normally distributed with a mean of 0.

Besides the theoretically appealing idea of allowing for a simultaneous generalization across both participants and items, mixed models offer two important statistical advantages [29], [46]. First, the main rationale for using mixed models is that they capture systematic variance in the data which, if ignored, distorts the estimation of the parameters of interest. For example, ignoring possible item effects can dramatically increase the probability of obtaining false positive results (up to empirical Type 1 error rates of.6 compared to the nominal.05; [29], Table 2). Second, whereas classical analysis only allows for a single observation per participant and cell of an experimental design, mixed models do not. In all experiments discussed so far there were exactly four trials (or replicates) for each participant and cell of the experimental design (e.g., there were four believable and valid syllogisms presented to each participant) which were aggregated prior to the analysis. This aggregation has the negative consequences that it decreases the precision of the statistical tests, specifically it reduces the precision with which the parameters were estimated.

**Table 2:** Results from the Bayesian Mixed Model Meta-Analysis.

#	Fixed Effects	BF	log(BF)
Participants			
1	believability	4.81 × 10^14^	33.81
2	validity	0.42	−3.18
3	validity+believability	2.00 × 10^13^	30.63
4	validity 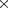 believability	4.52 × 10^12^	29.14
Participants plus slopes for believability			
5	none	1.39 × 10^10^	23.35
6	believability	1.06 × 10^14^	32.30
7	validity	5.86 × 10^08^	20.19
8	validity+believability	4.49 × 10^12^	29.13
9	validity 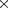 believability	1.15 × 10^12^	27.77
Participants plus slopes for believability 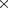 validity			
10	none	8.28 × 10^−8^	−16.31
11	believability	7.21 × 10^−4^	−7.23
12	validity	4.81 × 10^−9^	−19.15
13	validity+believability	4.19 × 10^−5^	−10.08
14	validity 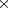 believability	2.44 × 10^−6^	−12.92
Participants and items			
15	none	7.23 × 10^48^	112.50
16	believability	3.23 × 10^48^	111.70
17	validity	2.32 × 10^47^	109.06
18	validity+believability	1.04 × 10^47^	108.26
19	validity 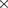 believability	2.17 × 10^46^	106.69
Participants and items plus slopes for believability			
20	none	2.78 × 10^50^	**116.15**
21	believability	1.72 × 10^50^	115.67
22	validity	8.93 × 10^48^	112.71
23	validity+believability	5.55 × 10^48^	112.24
24	validity 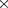 believability	1.34 × 10^48^	110.81
Participants and items plus slopes for believability 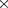 validity			
25	none	8.06 × 10^26^	61.95
26	believability	5.33 × 10^26^	61.54
27	validity	6.59 × 10^25^	59.45
28	validity+believability	4.36 × 10^25^	59.04
29	validity 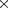 believability	3.47 × 10^24^	56.51

*Note.* All models are compared against the denominator model, M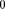, with only the random effect for participants and a fixed effect for experiment (BF for this model against the intercept only model: 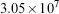). In addition to the effects mentioned, all models contain a fixed effect for experiment (potentially with slopes for believability and validity). The random-effects structure is given above each block of models. The analysis is based on the raw data from Klauer and Singmann ([20], Experiments 3 and 4) and Experiment 2 from the present manuscript. BF = Bayes factor. validity+believability = main effects for believability and validity. validity 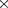 believability = main effects for believability and validity plus their interaction.

### Method

To reassess the evidence for a fluency mediated intuitive logic as proposed by MH, we performed a meta-analysis on the three experiments that obtained liking ratings for valid and invalid syllogisms in which the validity of the items was randomly assigned to the different contents (Experiments 3 and 4 of KS; Experiment 2 this manuscript). In total, 287 participants entered the analysis which was performed on the raw data (i.e., the four data points per participants and cell of the experimental design were not aggregated).

We performed the analysis by comparing a null or denominator model which only contained an overall intercept, random effects for participants, and a fixed effect for the experiment (with three levels, one for each experiment; Appendix S1 contains an alternative analysis in which experiment is treated as a random effect), designated as M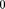, to models representing different hypotheses via Bayes factors obtained with the methods proposed by Rouder, Morey, and colleagues [21]. The models differed in both their random effects and fixed effects structure. Specifically, we implemented five different fixed effects structures: a model which contained no fixed effect over and above the effect of experiment which was present in all models (labeled “none” in Table 2), a model with only a main effect of believability, a model with only a main effect of validity, a model with both a main effect of validity and believability (labeled “validity 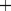 believability”), and finally a model with both main effects and an interaction of validity and believability (labeled “validity 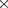 believability”). If MH's hypothesis were true, models containing a main effect of validity should be more likely (i.e., have a higher Bayes factor) than models without such an effect.

In terms of the random effects structure we manipulated two factors: (a) Whether or not the models included a random effect for the content of the conclusion or not (i.e., an item effect as discussed above). Note that English and German conclusions were treated as different items. (b) Whether the random effect had only random intercepts (i.e., no random slopes), additionally random slopes for believability, or additionally random slopes for believability, validity, and their interactions (if present, the item effect was only allowed to have random slopes for validity because items are nested within believability; a given conclusion can only be either believable or unbelievable). In total this amounted to six different random effects structures, the two versions of (a) times the three versions of (b). Furthermore, when there was a random slope for one of the fixed effects (believability or validity) across a random effect (item or participants), the corresponding slope for experiment was also included. The model which included random participant and item effects and random slopes for believability, validity, and their interactions corresponds to the model with maximal random effects structure recommended by Barr et al. [46].

In total we computed thirty different versions of the model, 29 of which were compared to the null or denominator model designated as M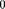. The analysis was performed using the BayesFactor package [42] for the statistical programming language R [38]. The R scripts for the analysis including the raw data are available in Appendix S2. We used the Laplace approximation to obtain estimates of the marginal likelihoods [21]. We used the 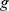-priors proposed by Rouder et al. [21] with a scale factor 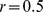 (for the fixed effects) which is recommended for small effect sizes.

### Results and Discussion

The results of the meta-analysis are presented in Table 2. A first inspection reveals that the random effects structure had an *extreme* influence on the Bayes factors in two ways. First, models without a random item effect (i.e., Models 1 to 14) were at least 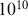 or 10 billion times less likely than models that included a random item effect. This is extreme evidence for heterogeneity among the items. Second, models that included random slopes for validity (i.e., Models 10 to 14 and 25 to 29) were also dramatically less likely than models that did not include such slopes indicating that there is evidence against heterogeneity of an effect of validity across participants and items.

In fact, there was evidence *against* any effect of validity. The model that provided the overall best account, Model 20, included a random effect for item and random slopes for believability but no further fixed effects, in particular no further effects of validity. The evidence for this model compared to the best model that included an effect of validity (i.e., Model 22) was *very strong*, 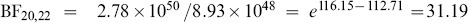. Furthermore, within all blocks with equal random effects structure the best model was always a model that did not include validity and the evidence in favor of the best model within each block compared to the best model that included an effect of validity was at least *strong*. The smallest Bayes factor of such a within-block comparison was 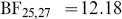.

The results regarding an effect of believability are somewhat more diverse. The overall best model did include random slopes for believability but no further fixed effect of it. However, the evidence for Model 20 compared to Model 21, which additionally included a fixed effect for believability, was only *anecdotal*: 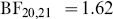. This pattern, slightly higher BFs for models without fixed effect for believability, is apparent for all three sets of models that include a random item effects. However, comparing the overall best model with the best model without any effect of believability (i.e., also without random believability slopes) “only” provided *very strong* evidence for an effect of believability, 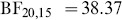. This indicates that there is evidence for heterogeneity of the believability effect across participants but slight evidence against a main effect of believability.

**Figure 2:**
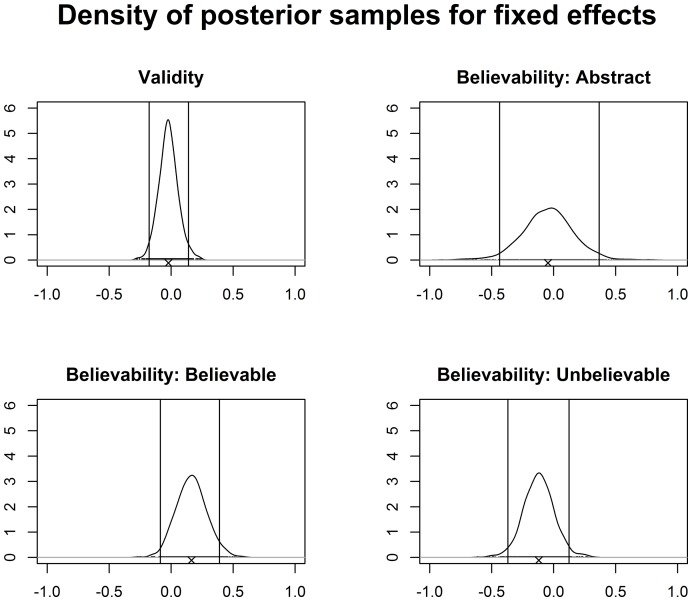
Density of 1000 posterior samples for the relevant fixed effects of Model 29. The 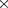 below each plot depicts the mean. The 95% credible intervals are depicted by two vertical lines. The tiny black points at the 0 line show the position of the samples. The plot for validity shows the fixed effect for valid inferences, the corresponding plot for invalid inferences would be mirrored at 0.

To further inspect the results we obtained 1000 samples for each parameter from the posterior distribution of the largest model (Model 29). We chose this model as it included all relevant fixed and random effects (i.e., it realized the maximal random effects structure [46]) and hence estimation of all effects should have been the most precise. The densities and 95% credible intervals of the posterior samples of the relevant fixed effects parameters are displayed in Figure 2 which shows the same pattern as the BF analysis. The mean fixed effect parameter for validity was virtually 0 (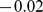) and the credible interval very narrow and included 0. For the fixed effect parameters for believability the pattern is somewhat different: the credible intervals are wider, although all include 0. The mean parameter values for believable and unbelievable items were, in line with prior expectations, positive for believable items, 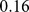, and negative for unbelievable items, 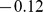.

**Figure 3:**
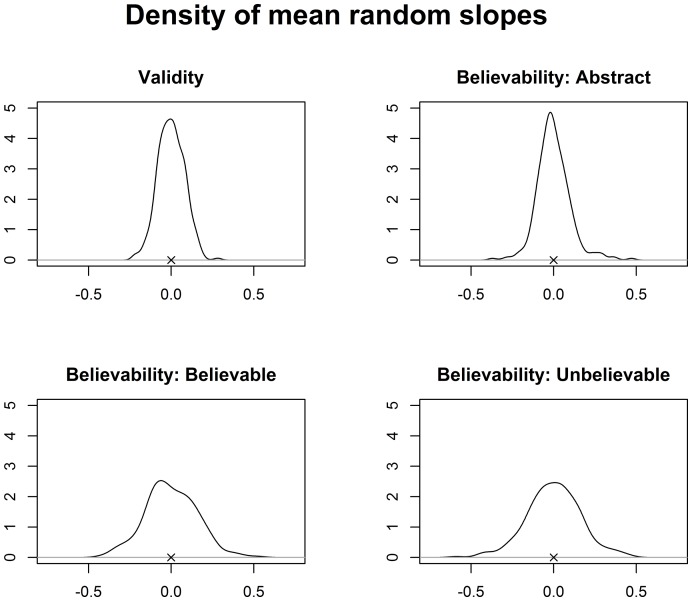
Density of mean individual parameter estimates for the relevant random participant slopes of Model 29. The 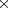 depicts the mean. The plot for validity shows the random slopes for valid inferences, the corresponding plot for invalid inferences would be mirrored at 0.

To assess the magnitude of the random slopes, we calculated the mean parameter values for each individual slope parameter (i.e., one per participant or item) from the posterior samples. The densities of those mean parameter values for the participant slopes are displayed in Figure 3 and again replicate the BF analysis. For validity the density is rather narrow (SD = 0.08) indicating little heterogeneity in the validity effect. In contrast, for believability there is considerable heterogeneity in the random slopes, at least for believable and unbelievable items (both SDs = 0.16, SD for abstract items = .10).

To test the robustness of the results we repeated the analysis reported here with the following changes: In one analysis the factor experiment was treated as a random effect. In another analysis we changed the scaling factor of the 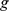-priors for the fixed effects to the very small value 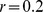. Neither of the changes affected the overall pattern of results with one exception: When the 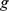-priors were very small, models with only a fixed effect for believability had (at least slightly) larger BFs than the model with the same random effects structure but without any fixed effects. The corresponding results tables can be found in Appendix S1.

As suggested by an anonymous reviewer, we also estimated an additional non-Bayesian mixed model analysis of the data. This analysis, however, only used the believable and unbelievable trials as there were no abstract trials in Experiment 2 (this manuscript). In other words, the factor “believability” had 3 levels (believable, unbelievable, & abstract) for most of the data (and in the Bayesian meta-analysis), but not for Experiment 2. Because of these differences in the experiments, a joint analysis of all data in a classical linear model approach required dropping the abstract trials. In accordance with the results from the Bayesian analysis, the classical analysis also revealed no effect of validity, 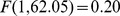, 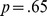. The difference between invalid and valid trials was estimated to be 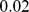, whereas it was estimated to be 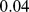 in the Bayesian analysis. Similar to when using a very narrow prior of 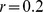, there was evidence for an effect of believability, 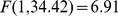, 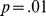. However, given our sample size this evidence can be considered somewhat weak (see also [47]). The difference between believable and unbelievable items was estimated to be 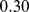, which agrees with the estimate from the Bayesian analysis which was 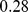. No further effects reached significance, all 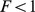. More details on this analysis and a full results table can be found in Appendix S1.

Taken together, this meta-analysis provided three main results: there was very strong evidence against the hypothesis of a fluency mediated intuitive logic, there was extreme evidence for item heterogeneity, and there was strong evidence for heterogeneity of an effect of believability of the conclusion but hardly any evidence for an overall effect of believability.

## General Discussion

The results presented in this paper allow one to make a stronger inference concerning the evidence for a fluency mediated logic in the paradigm developed by MH: When controlling for effects of the content of the conclusion, as done in three experiments with 287 participants, there is not only no evidence for an influence of validity of the syllogism on the liking ratings of the conclusion, but there is very strong evidence against such an effect as expressed in the Bayesian analysis.

The inference drawn here is not only stronger, but also extends the results of KS in another way. The new experiments presented here adopted some procedural changes that should have made it more likely to observe intuitive logic as the context provided by the task more strongly suggested inferential reasoning. Additionally, the presentation of the syllogism was self-paced, which was also the case in most of the experiments reported by MH. As we still did not find evidence for an intuitive logic, these limitations to the results discussed by KS do not apply anymore. However, it should be noted that we only used rather complicated inferences and our results cannot be extended to really simple syllogisms which were also used by MH or [48].

Furthermore, our results do not entail that fluency or other transient affective or cognitive effects do not affect how individuals rate the likeability of sentences. For example, in MH's Experiment 3 participants were listening to classical music while rating the likeability of the conclusions and, employing a misattribution paradigm, when led to believe that this music would influence their emotional reaction, showed a reduced “validity” effect (as we have shown here this effect was not a validity effect but due the affective connotations of the content of the conclusions). This suggests that fluency experiences and how they are attributed play a role in liking ratings of the conclusion. In fact, it is well known that factors such as grammatical complexity, readability, familiarity, or prior exposure have an impact on affective reactions toward sentences that is mediated by effects of fluency (see [49] for a current overview). What we have shown here is that the logical form is no source of perceived fluency with which the syllogisms are parsed.

Additionally, our analysis showed that participants did not respond randomly in the task. The Bayesian meta-analysis provides *extreme* evidence for participant and even more strongly for item effects. In fact, a simple model with only random intercepts for participants and items (plus a fixed effect of experiment), Model 15, is among the best models overall. This model is only further improved by considering believability. However, while there is heterogeneity of the believability effects among participants (i.e., higher BFs for models with random believability slopes, see also Figure 3), there is hardly any evidence for an overall main effect of believability. Additionally, the results of Experiments 1 and 2 compared to the results of KS indicate that affective connotations of the materials, which obviously differed between a German and an English sample, are an important predictor of participants' liking ratings.

We consider the fact that the specific items played such an important role as one of the additional take home messages of our study as item effects are rarely taken into account in reasoning research. It is well known that a failure to do so can lead to dramatically distorted statistics [29] which is also apparent in our analysis. When properly controlling for participants and item effects and employing an appropriate random effects structure [46] our analysis revealed that there is even slight evidence against a main effect of believability. Consequently, we highly recommend to explicitly model item effects whenever possible. Either, as was done here, by explicitly including item as a random effect with the appropriate random effects structure (i.e., on the same level as participant, so creating crossed random effects). Alternatively, especially when the number of items is small, item can be included as a fixed effect into the model ([50] for an example).

Our analysis revealed one important aspect to consider when performing a Bayesian mixed model analysis. When estimating a non-Bayesian mixed model, Barr and colleagues [46] made a very strong point for always employing the maximal random effects structure. Their main rationale for this recommendation is (a) that omitting a relevant random slope produces more biased estimates and generates more wrong conclusions compared to introducing a superfluous random slope and (b) that there does not exist a generally applicable data-driven approach (e.g., forward versus backward selection) to obtain the optimal random effects structure. Whereas argument (a) holds for Bayesian and non-Bayesian mixed models, argument (b) is mainly a problem for non-Bayesian analysis – although some frequentist testing approaches have been discussed [51] – as Bayes factors employ a sort of automatic Occam's razor and punish models with overly complex random effects structures. Hence, although we agree with Barr et al. that the maximal random effects structure should always be among the compared models, it makes sense to include other random effects structures as well. Bayes factors can help to determine the optimal random effects structure thereby uncovering which effects differ among the random effects in the model. The fact that Bayes factors are consistent when comparing across different models makes this approach even more convenient and appealing. As our analysis has shown, it also makes sense to include random slopes without the corresponding fixed main effects, as both cover different aspects of the data.

Finally, we would like to go beyond our data and address the idea of an intuitive logic in general. At their core, the accounts of MH and De Neys [13] share a similar notion of intuitive logic. However, both accounts were developed independently and are differently motivated. More specifically, employing a range of tasks (e.g., base-rate neglect [15] or the bat-and-ball problem [52]) and measures (e.g., eye- and gaze tracking [15] or memory probing [53]) De Neys and colleagues observed that participants, although giving the heuristic or biased response, seemed to show some sensitivity to their errors. Based on these findings De Neys hypothesized that individuals are able to intuitively detect the normatively correct response according to standard logic or according to standard probability theory (e.g., [13], pp. 28 and p. 35). This stands in stark contrast with the majority of contemporary theories on reasoning and decision making according to which theories cannot be built around standard logic and probability theory as normative systems [6], [7], [54]–[56]. Given this discrepancy, we think that the evidence provided by proponents of intuitive logic needs to be extraordinary strong – “extraordinary claims require extraordinary evidence” ([57], p. 429) – and proponents should take care to rule out alternative explanations, using, for example, procedural techniques as implemented here (randomization of contents) and by KS (use of *pseudo-problems*).

## Supporting Information

Appendix S1
**Alternative analyses of the meta-analysis.** The document contains results tables of four different versions of the meta-analysis reported in the manuscript: (a) the original analysis as reported in the manuscript, (b) an alternative analysis in which experiment is treated as random effect (as compared to as a fixed effect as was done in the original analysis), (c) an alternative analysis with 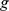-priors of 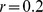, and (d) a non-Bayesian mixed model analysis of the data.(PDF)

Appendix S2 **Data and analysis script for the meta-analysis.**
(ZIP)
