# Supplementary material for: Intuitive Logic Revisited: New Data and a Bayesian Mixed Model Meta-Analysis
Source: PLoS One. 2014 Apr 22;9(4):e94223. doi: 10.1371/journal.pone.0094223 (PMC3995688; doi:10.1371/journal.pone.0094223)
Supplement: Appendix S1 — Alternative analyses of the meta-analysis. The document contains results tables of four different versions of the meta-analysis reported in the manuscript: (a) the original analysis as reported in the manuscript, (b) an alternative analysis in which experiment is treated as random effect (as compared to as a fixed effect as was done in the original analysis), (c) an alternative analysis with -priors of , and (d) a non-Bayesian mixed model analysis of the data. (PDF) [file pone.0094223.s001.pdf]

Intuitive Logic Revisited: New Data and a Bayesian Mixed Model Meta-Analysis - Supplemental Material

Henrik Singmann Karl-Christoph Klauer David Kellen

Albert-Ludwigs-Universität Freiburg

Contact Henrik Singmann (henrik.singmann@psychologie.uni-freiburg.de) in case of questions.

Supplemental Results Tables

This document contains alternative analyses for the meta-analysis reported in the main paper. More specifically, the document contains the results tables of different versions of the meta-analysis. Specifically the following analyses are presented:

1. The original meta-analysis as reported in the paper with the denominator model, M_0, having a random effect for participants but a fixed effect for experiment (BF for this model against the intercept only model: 3.05×10^7). The g-priors had the scale factor $r = 0.5$ (for the fixed effects) which is recommended for small effect sizes.

2. An alternative meta-analysis with the denominator model, M_0, having random effects for both participants and experiment (BF for this model against the intercept only model: 7.82×10^6). The g-priors had the scale factor $r = 0.5$ (for the fixed effects) which is recommended for small effect sizes.

3. An alternative meta-analysis with the denominator model, M_0, as in the original analysis having a random effect for participants but a fixed effect for experiment (BF for this model against the intercept only model: 1.80×10^8). Here, the g-priors had the very low scale factor $r = 0.2$ (for the fixed effects).

4. Finally, this document also contains a non-Bayesian Mixed Model meta-analysis.

For all tables applies:

The random-effects structure is given above each block of models.
The analysis is based on the raw data from Klauer and Singmann (in press, Experiments 3 and 4) and Experiment 2 from the present manuscript.
BF = Bayes factor
validity + believability = main effects for believability and validity
validity × believability = main effects for believability and validity plus their interaction.

Table 1: Original Results from the Bayesian Mixed Model Meta-Analysis

#	Fixed Effects	BF	log(BF)
	Participants		
1	believability	4.81×10^{14}	33.81
2	validity	0.42	-3.18
3	validity + believability	2.00×10^{13}	30.63
4	validity × believability	4.52×10^{12}	29.14
	Participants plus slopes for believability		
5	none	1.39×10^{10}	23.35
6	believability	1.06×10^{14}	32.30
7	validity	5.86×10^{08}	20.19
8	validity + believability	4.49×10^{12}	29.13
9	validity × believability	1.15×10^{12}	27.77
	Participants plus slopes for believability × validity		
10	none	8.28×10^{-8}	-16.31
11	believability	7.21×10^{-4}	-7.23
12	validity	4.81×10^{-9}	-19.15
13	validity + believability	4.19×10^{-5}	-10.08
14	validity × believability	2.44×10^{-6}	-12.92
	Participants and items		
15	none	7.23×10^{48}	112.50
16	believability	3.23×10^{48}	111.70
17	validity	2.32×10^{47}	109.06
18	validity + believability	1.04×10^{47}	108.26
19	validity × believability	2.17×10^{46}	106.69
	Participants and items plus slopes for believability		
20	none	$\mathbf{2.78 \times 10^{50}}$	**116.15**
21	believability	1.72×10^{50}	115.67
22	validity	8.93×10^{48}	112.71
23	validity + believability	5.55×10^{48}	112.24
24	validity × believability	1.34×10^{48}	110.81
	Participants and items plus slopes for believability × validity		
25	none	8.06×10^{26}	61.95
26	believability	5.33×10^{26}	61.54
27	validity	6.59×10^{25}	59.45
28	validity + believability	4.36×10^{25}	59.04
29	validity × believability	3.47×10^{24}	56.51

Note. All models are compared against the denominator model, M_0, with only the random effect for participants and a fixed effect for experiment (BF for this model against the intercept only model: 3.05×10^7). In addition to the effects mentioned, all models contain a fixed effect for experiment (potentially with slopes for believability and validity). The g-priors had the scale factor $r = 0.5$ (for the fixed effects).

Table 2: Alternative Results with Experiment as Random Effect

#	Fixed Effects	BF	log(BF)
	Participants		
1	believability	4.82×10^{14}	33.81
2	validity	4.14×10^{-02}	-3.18
3	validity + believability	2.01×10^{13}	30.63
4	validity × believability	4.53×10^{12}	29.14
	Participants plus slopes for believability		
5	none	4.77×10^{13}	31.50
6	believability	1.69×10^{13}	30.46
7	validity	2.02×10^{12}	28.33
8	validity + believability	7.13×10^{11}	27.29
9	validity × believability	1.83×10^{11}	25.93
	Participants plus slopes for believability × validity		
10	none	2.51×10^{-07}	-15.20
11	believability	8.90×10^{-08}	-16.23
12	validity	1.16×10^{-07}	-15.97
13	validity + believability	4.13×10^{-08}	-17.00
14	validity × believability	1.30×10^{-08}	-18.16
	Participants and items		
15	none	7.39×10^{48}	112.52
16	believability	3.30×10^{48}	111.72
17	validity	2.37×10^{47}	109.08
18	validity + believability	1.06×10^{47}	108.28
19	validity × believability	2.21×10^{46}	106.71
	Participants and items plus slopes for believability		
20	none	$\mathbf{8.05 \times 10^{49}}$	**114.91**
21	believability	2.92×10^{49}	113.90
22	validity	2.59×10^{48}	111.48
23	validity + believability	9.39×10^{47}	110.46
24	validity × believability	2.26×10^{47}	109.04
	Participants and items plus slopes for believability × validity		
25	none	3.78×10^{23}	54.29
26	believability	1.38×10^{23}	53.28
27	validity	1.76×10^{23}	53.53
28	validity + believability	6.45×10^{22}	52.52
29	validity × believability	2.08×10^{22}	51.39

Note. All models are compared against the denominator model, M_0, with random effect for participants and experiment (BF for this model against the intercept only model: 7.82×10^6). In addition to the effects mentioned, all models contain a random effect for experiment (potentially with slopes for believability and validity). The g-priors had the scale factor $r = 0.5$ (for the fixed effects).

Table 3: Alternative Results with $r = 0.2$

#	Fixed Effects	BF	log(BF)
	Participants		
1	believability	1.30×10^{15}	34.80
2	validity	1.02×10^{-01}	-2.28
3	validity + believability	1.34×10^{14}	32.53
4	validity × believability	1.09×10^{14}	32.32
	Participants plus slopes for believability		
5	none	5.94×10^{10}	24.81
6	believability	1.78×10^{15}	35.12
7	validity	6.18×10^{09}	22.54
8	validity + believability	1.85×10^{14}	32.85
9	validity × believability	1.71×10^{14}	32.77
	Participants plus slopes for believability × validity		
10	none	1.19×10^{-05}	-11.34
11	believability	4.13×10^{-01}	-0.88
12	validity	1.52×10^{-06}	-13.39
13	validity + believability	5.30×10^{-02}	-2.94
14	validity × believability	1.13×10^{-02}	-4.48
	Participants and items		
15	none	7.47×10^{48}	112.53
16	believability	9.34×10^{48}	112.76
17	validity	5.93×10^{47}	110.00
18	validity + believability	7.41×10^{47}	110.22
19	validity × believability	5.63×10^{47}	109.95
	Participants and items plus slopes for believability		
20	none	2.74×10^{51}	118.44
21	believability	$\mathbf{3.14 \times 10^{51}}$	**118.57**
22	validity	2.18×10^{50}	115.91
23	validity + believability	2.50×10^{50}	116.04
24	validity × believability	2.15×10^{50}	115.90
	Participants and items plus slopes for believability × validity		
25	none	2.87×10^{29}	67.83
26	believability	3.40×10^{29}	68.00
27	validity	5.58×10^{28}	66.19
28	validity + believability	6.61×10^{28}	66.36
29	validity × believability	1.81×10^{28}	65.06

Note. All models are compared against the denominator model, M_0, with random effect for participants and experiment (BF for this model against the intercept only model: 1.80×10^8). In addition to the effects mentioned, all models contain a random effect for experiment (potentially with slopes for believability and validity). The g-priors had the scale factor $r = 0.2$ (for the fixed effects).

Non-Bayesian Analysis

We also analyzed the data with a non-Bayesian mixed model. Specifically, we estimated a model with fixed effects for validity × believability × experiment while also estimating the maximal random effects structure as recommended by Barr, Levy, Scheepers, and Tily (2013), which corresponds to the random effects structure of models 25 to 29 in the main manuscript. As discussed in more detail in the main manuscript, we only included data from trials with believable and unbelievable conclusions (i.e., no abstract conclusions). In addition to the parameters estimated for the Bayesian Mixed Models we also estimated the correlations between the random intercepts and corresponding random slopes as such parameters are at the moment not implemented in the `BayesFactor` package (Richard Morey, personal communication, January 8, 2014). We estimated the model with restricted maximum-likelihood using `lme4` (Bates, Maechler, Bolker, & Walker, 2013) for the statistical programming language `R` (R Core Team, 2013).

To estimate the significance of fixed effects, we compared the full model with a model in which the effect of interest was excluded using the methods implemented in `afex` (Singmann, 2013). Denominator degrees of freedom were based on the Kenward-Rogers approximation for degrees of freedom as implemented in `pbkrtest` (Halekoh & Højsgaard, 2013). The results of this analysis are presented in the following table:

Effect	Parameter	F	*df*	F-scaling	p
(Intercept)	3.18	2943.00	1, 40.14	1.00	$<$ .001
validity	-0.01	0.20	1, 62.05	1.00	.65
believability	0.15	6.91	1, 34.42	1.00	.01
experiment	[0.06]	0.47	2, 89.93	0.99	.63
validity × believability	-0.02	0.90	1, 59.12	1.00	.35
validity × experiment	[0.01]	0.02	2, 119.25	1.00	.98
believability × experiment	[0.03]	0.11	2, 79.10	0.99	.90
validity × believability × experiment	[0.02]	0.09	2, 114.98	1.00	.92

Note. Values in squared brackets [ ] represent the estimated maximum difference for the three experiments.

References

Barr, D. J., Levy, R., Scheepers, C., & Tily, H. J. (2013). Random effects structure for confirmatory hypothesis testing: keep it maximal. *Journal of Memory and Language*, *68*(3), 255–278. doi:10.1016/j.jml.2012.11.001

Bates, D., Maechler, M., Bolker, B., & Walker, S. (2013). *Lme4: linear mixed-effects models using eigen and s4*. R package version 1.1-0. http://lme4.r-forge.r-project.org/.

Halekoh, U. & Højsgaard. (2013). *Pbkrtest: parametric bootstrap and kenward roger based methods for mixed model comparison*. R package version 0.3-5.1. http://people.math.aau.dk/ soren[illegible]

R Core Team. (2013). *R: a language and environment for statistical computing*. http://www.R-project.org/. Vienna, Austria: R Foundation for Statistical Computing.

Singmann, H. (2013). *Afex: analysis of factorial experiments*. R package version 0.6-77/r77. http://www.psychologie.uni-freiburg.de/Members/singmann/R/afex.
